# Assessment of Plasma and Cerebrospinal Fluid Biomarkers in Different Stages of Alzheimer’s Disease and Frontotemporal Dementia

**DOI:** 10.3390/ijms24021226

**Published:** 2023-01-08

**Authors:** Lourdes Álvarez-Sánchez, Carmen Peña-Bautista, Laura Ferré-González, Angel Balaguer, Miguel Baquero, Bonaventura Casanova-Estruch, Consuelo Cháfer-Pericás

**Affiliations:** 1 Alzheimer Disease Research Group, Instituto de Investigación Sanitaria La Fe, 46026 Valencia, Spain; 2 Faculty of Mathematical Sciences, University of Valencia, 46100 Burjassot-Valencia, Spain; 3 Division of Neurology, University and Polytechnic Hospital La Fe, 46026 Valencia, Spain

**Keywords:** Alzheimer’s disease, frontotemporal dementia, plasma, biomarker, SIMOA, diagnosis

## Abstract

Alzheimer’s disease (AD) is the primary type of dementia, followed by frontotemporal lobar degeneration (FTLD). They share some clinical characteristics, mainly at the early stages. So, the identification of early, specific, and minimally invasive biomarkers is required. In this study, some plasma biomarkers (Amyloid β42, p-Tau181, t-Tau, neurofilament light (NfL), TAR DNA-binding protein 43 (TDP-43)) were determined by single molecule array technology (SIMOA^®^) in control subjects (*n* = 22), mild cognitive impairment due to AD (MCI-AD, *n* = 33), mild dementia due to AD (*n* = 12), and FTLD (*n* = 11) patients. The correlations between plasma and cerebrospinal fluid (CSF) levels and the accuracy of plasma biomarkers for AD early diagnosis and discriminating from FTLD were analyzed. As result, plasma p-Tau181 and NfL levels correlated with the corresponding CSF levels. Additionally, plasma p-Tau181 showed good accuracy for distinguishing between the controls and AD, as well as discriminating between AD and FTLD. Moreover, plasma NfL could discriminate dementia-AD vs. controls, FTLD vs. controls, and MCI-AD vs. dementia-AD. Therefore, the determination of these biomarkers in plasma is potentially helpful in AD spectrum diagnosis, but also discriminating from FTLD. In addition, the accessibility of these potential early and specific biomarkers may be useful for AD screening protocols in the future.

## 1. Introduction

Alzheimer’s disease (AD) is the primary type of dementia, and its incidence is expected to increase in the following years. It shows similar characteristics with frontotemporal lobar degeneration (FTLD), which represents a group of heterogeneous disorders and may be the second cause of dementia in the population under 65 years [1,2]. Nowadays, differential AD diagnosis is based on cerebrospinal fluid (CSF) biomarkers [3,4,5,6]. However, CSF sampling is invasive and sometimes cannot be applied because of the patient’s characteristics or logits circumstances. Furthermore, it cannot be used as a screening method. For these reasons, plasma biomarkers have been investigated in recent years, finding that the classical biomarkers (amyloid-β42 (Aβ42), t-Tau, p-Tau181) were found at low concentrations, preventing their detection [7].

Identifying plasma biomarkers to diagnose neurodegenerative diseases could improve the accurate and specific detection of the pathology, discriminating between AD and other clinical pathologies [8]. In addition, the use of plasma biomarkers could improve the early diagnosis of AD, even years before the first symptoms appear [9]. Additionally, it could be helpful for a better knowledge of the physiopathological mechanisms involved in first stages of these diseases [10,11].

Recent clinical trials’ results showed the need to identify the earliest stages of the disease, where the neurodegeneration remains in a mild stage. More accessible and less invasive potential biomarkers are needed, and more accessible biomarkers could help to monitor neurodegeneration [12]. In recent years, new high sensitivity techniques, such as mass spectrometry [3] and digital ELISA, have been developed, and it could be possible to determine compounds at low concentrations, opening new possibilities [12].

The core AD biomarkers for amyloidosis (Aβ42 and ratio Aβ42/Aβ40) have been measured in plasma for the diagnosis of AD, even in the early stages [13,14,15]. A previous study showed the consistency of the plasma ratio Aβ42/Aβ40 as a predictor of AD [16], and plasma p-Tau181 has been studied as biomarker for the disease [17,18,19]. Moreover, these recent studies pointed to plasma p-Tau181 and other hyperphosphorylated Tau (p-Tau231, p-Tau217) as the most sensitive plasma biomarkers for AD diagnosis [18,19], even in the early stages [20,21]. In general, individual plasma biomarkers have shown good accuracy for AD diagnosis, which could be improved by the simultaneous combination of some plasma biomarkers [22,23,24,25]. The neurofilament light chain (NfL) is another molecule that has been analyzed in the context of AD as a progression biomarker [26] or predictor for dementia, even more helpful in other pathologies, such as FTLD [27,28]. Different works pointed out the potential use of these biomarkers mentioned, which could change the clinical guides in the following years, with earlier and higher accessible diagnosis [29,30,31,32].

Other potential AD plasma biomarkers could be related to abnormal astroglia activation response in neurodegenerative diseases (e.g., GFAP) [33,34], triggering receptor expressed on myeloid cells 2 (TREM2)) [35]. Additionally, TAR DNA-binding protein 43 (TDP-43) has been determined to evaluate its role in the neurodegeneration pathway. Actually, the presence of phosphorylated TDP-43 accumulated intraneuronal in regions such as the amygdala, at first, and later extended to the hippocampal regions and the rest of the brain parenchymal, appearing in the entity called the TDP-43 encephalopathy neuropathological change (LATE-NC), which is related to aging [36]. Additionally, anatomopathological studies of the brain showed TDP-43 inclusions in up to 57% of patients with AD with limbic distribution predominant [37]. This finding suggests that TDP-43 inclusions could have a role in the development of the disease or could mean concomitance with others neurodegenerative pathologies [38].

Regarding specific diagnosis, some plasma biomarkers could be able to differentiate AD from other neurodegenerative diseases [39]. In this sense, mainly the discrimination between AD and FTLD has been evaluated [40]; specifically, analyzing potential progression markers [41].

In this sense, the present study aims to analyze the combination of different plasma biomarkers (Aβ40, Aβ42, p-Tau181, t-Tau, NfL, and TDP-43) to improve the early and specific AD diagnosis. Additionally, the range of normal and pathological plasma concentrations for each compound will be determined, and their correlations with the corresponding CSF levels will be evaluated.

## 2. Results

### 2.1. Demographic and Clinical Description of Participants

The demographic and clinical characteristics are summarized in Table 1. There were not statistically significant differences for sex among groups. The differences in age were statistically significant among groups (*p* < 0.03), but analyzing between them, only statistically significant differences were obtained for FTLD vs. controls and vs. dementia-AD. As expected, the clinical variables showed statistically significant differences between groups for neuropsychological tests and CSF biomarkers.

CSF Aβ42 levels were lower in the AD group (MCI-AD, dementia-AD) than in the control group. Additionally, the FTLD group showed lower levels than the control group, but higher than the AD-group. The CSF t-Tau values were higher in the AD group (MCI-AD 551 pg/mL, dementia-AD 978 pg/mL) than in the control group (235 pg/mL) and in the FTLD group (351 pg/mL). CSF p-Tau181 levels were lower in the control group (49 pg/mL) than in the AD-groups (MCI-AD 92 pg/mL, dementia-AD 175 pg/mL), but the lowest values were obtained in FTLD group (42 pg/mL). CSF NfL levels were higher for the FTLD group (1607 pg/mL) than for the AD-groups (MCI-AD 952 pg/mL, dementia-AD 1408 pg/mL), and the control group showed the lowest levels (590 pg/mL).

### 2.2. Correlations between Plasma and CSF Biomarkers Levels

Correlation results between plasma and CSF biomarkers levels are shown in Table 2. Positive correlations were observed for p-Tau181 (r = 0.649, *p* < 0.01) and NfL (r = 0.86, *p* < 0.01). In addition, plasma p-Tau181 correlated with CSF t-Tau (r = 0.611, *p* < 0.01) and CSF ratio t-Tau/Aβ42 (r = 0.605, *p* < 0.01); and plasma ratio Aβ42/Aβ40 and CSF Aβ42 levels (r = 0.277, *p* = 0.01) showed a significant correlation. In contrast, negative correlations were obtained between the plasma Aβ42/Aβ40 ratio and CSF t-Tau (r = −0.27, *p* = 0.02), CSF p-Tau181 (r = −0.297, *p* < 0.01), and CSF ratio t-Tau/Aβ42 (r = −0.497, *p* > 0.01). There were no correlations obtained between Aβ42 plasma levels and Aβ42 CSF levels, nor between t-Tau plasma and CSF levels. Additionally, TDP-43 plasma levels did not correlate with CSF levels. Although some significant correlations were observed between the plasma ratio Aβ42/Aβ40 and some CSF biomarkers, no significant correlations were observed between the plasma ratio t-Tau/Aβ42 and CSF biomarkers.

### 2.3. Plasma Biomarkers Levels in Participants Groups

The results obtained for the plasma biomarkers determined in each participant group are summarized in Table 3. As can be seen, statistically significant differences were obtained for p-Tau181, NfL, and ratio Aβ42/Aβ40 among all the groups (see *p*-value Kruskal–Wallis). For p-Tau181, the highest plasma levels were obtained in the dementia-AD group, followed by the MCI-AD group and the HC group, which showed the lowest levels. Additionally, analyzing the differences between groups, statistically significant differences were obtained between MCI-AD and HC, MCI-AD vs. FTLD, dementia-AD vs. HC, and dementia-AD vs. FTLD; however, the differences between MCI-AD and dementia-AD were not significant. For NfL, the highest levels were obtained in the dementia-AD group, followed by the FTLD group, and the lower levels were measured in the HC group. Moreover, statistically significant differences were obtained for NfL between HC and dementia-AD groups, HC vs. FTLD, and MCI-AD vs. dementia-AD. The differences between dementia-AD and the FTLD group were not significant, nor were differences between HC and MCI-AD. For the ratio Aβ42/Aβ40, statistically significant differences were obtained for MCI-AD vs. HC and FTLD.

For Aβ42, lower plasma levels were obtained in the AD groups (MCI and dementia) than in HC and FTLD groups, but no statistically significant differences were obtained. For t-Tau, higher plasma levels were obtained in the AD group, compared to the FTLD and HC groups, but these differences were not statistically significant.

In addition, no significant differences were obtained for t-Tau/Aβ42 ratio, nor for TDP-43, which showed the highest levels in the dementia-AD group, followed by the FTLD group and the lowest levels in the HC group.

**Figure 1:**
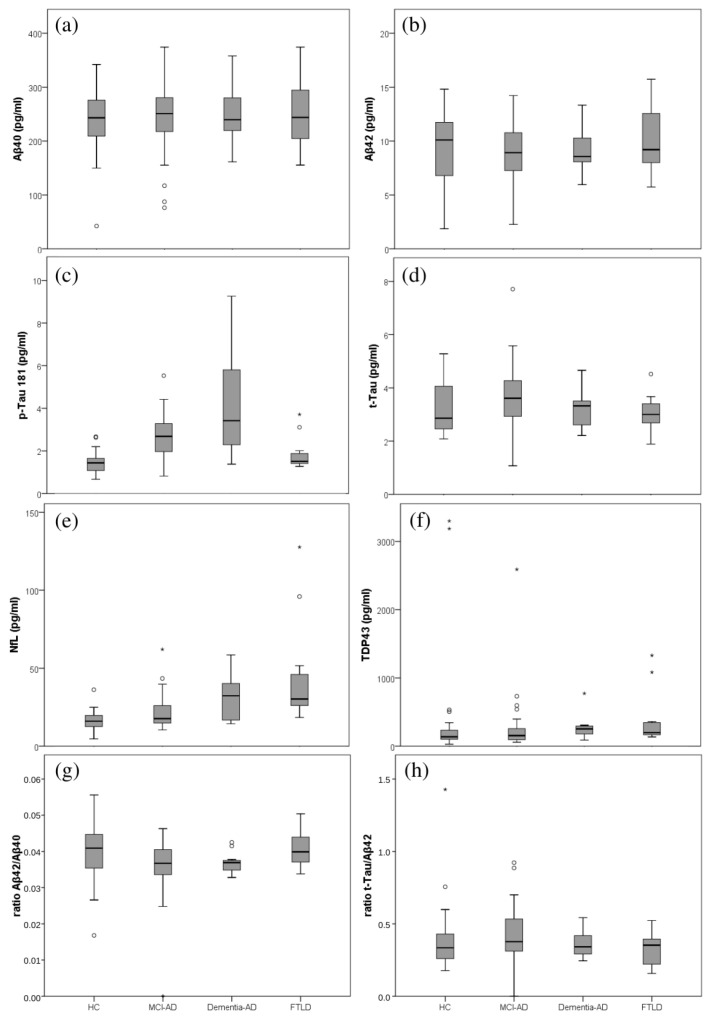
Box plots representing the plasma levels in the different participants groups (healthy controls (HC), MCI-AD, dementia-AD, FTLD). (**a**) Aβ40; (**b**) Aβ42; (**c**) p-Tau181; (**d**) t-Tau; (**e**) NfL; (**f**) TDP-43 (**g**) ratio Aβ42/Aβ40; (**h**) ratio t-Tau/Aβ42.

As can be seen, Figure 1 depicts the box plots for each plasma biomarker in the participants groups. For Aβ40, the plasma levels were homogeneous for all the groups. The Aβ42 levels were lower in the AD group, compared to HC and FTLD groups, while p-Tau181 levels were higher in the AD group (MCI-AD, dementia-AD). For NfL, the plasma levels increased along the AD stages.

### 2.4. Multivariant Analysis in AD Diagnosis Model’s Development

From the multivariant analysis, some PLS models were developed using the plasma biomarkers as predictor variables (Aβ42, Aβ40, t-Tau, p-Tau181, NfL, TDP-43), and the participants group as a response variable, to detect early and specific AD. These models showed 1–2 principal components (PC).

Then, a receiver operating characteristic (ROC) curve analysis was performed to estimate the diagnosis potential of this panel of plasma biomarkers in each developed model. The results obtained for each PLS model are summarized in Table 4. Among them, it is important to highlight the model discriminating AD patients, since the early stages (MCI-AD, mild dementia-AD), from HC subjects, and it showed satisfactory accuracy (AUC 0.809), as well as sensitivity (73.3%), specificity (86.4%), and PPV (91.7%). Similarly, the model discriminating HC vs. MCI-AD showed satisfactory accuracy (AUC 0.802), as well as sensitivity (69.7%) and specificity (86.4%). In addition, the model discriminating MCI-AD patients from FTLD subjects showed high accuracy (AUC 0.813), reflecting its potential capacity in specific AD diagnoses, as well as the model discriminating AD patients (MCI-AD, mild dementia-AD) from FTLD patients (AUC 0.796, sensitivity 62.2%, specificity 100%).

**Figure 2:**
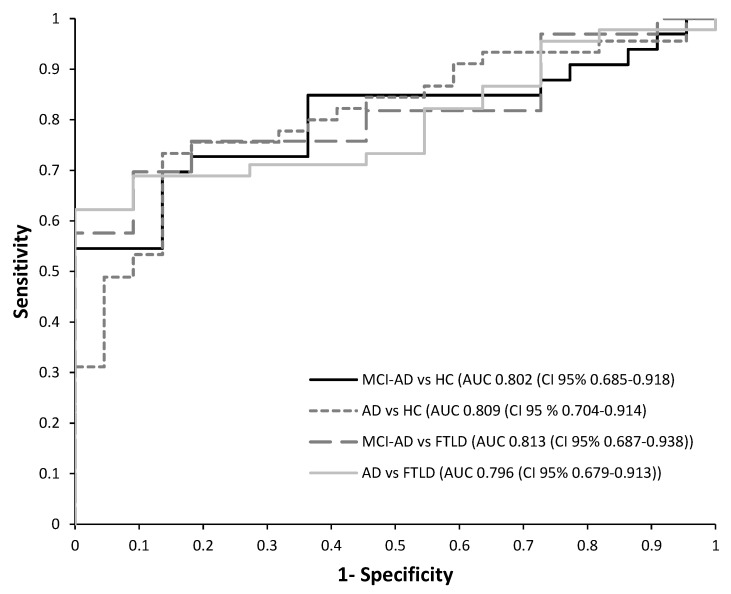
Receiver operating characteristic (ROC) curves for the PLS models developed from plasma biomarkers levels. (1) HC vs. MCI-AD; (2) HC vs. AD (MCI-AD + dementia-AD); (3) MCI-AD vs. FTLD; (4) AD (dementia-AD + MCI-AD) vs. FTLD.

Figure 2 shows the ROC curves obtained for the different developed models. In these models, the p-Tau181 and NfL variables showed high discriminating capacity, as can be seen from regression coefficients (Table 5).

In the developed diagnosis models, the predictor variables were combined using the following equation, in order to calculate the individual probability of suffering from AD. The corresponding corrected coefficients for each model equation are shown in Table 6.

Pr(AD) = a + b[Aβ40] + c[p-Tau181] + d[Aβ42] + e[t-Tau] + f[NfL ]+ g[TDP-43]

### 2.5. Plasma Biomarkers and Clinical Variables Correlation

The correlations between plasma biomarkers levels and clinical variables (neuropsychological performance, age) were evaluated. As can be seen in Table 7, the NfL levels were positively correlated with the CDR (clinical dementia rating scale) scores (*p* < 0.01) and negatively correlated with the MMSE (mini-mental state examination scale) scores (*p* < 0.01). Additionally, the *p*-Tau181 levels were negatively correlated with the MMSE scores (*p* < 0.01). Moreover, significant correlations were found between RBANS-DM (repeatable battery for the assessment of neuropsychological status) and t-Tau (*p* < 0.01), ratio t-Tau/Aβ42 (*p* < 0.01), and TDP-43 (*p* < 0.01). In general, a negative correlation was obtained between the Aβ42/Aβ40 ratio and age (*p* < 0.01).

Analyzing these correlations in the different groups, it was observed that in the HC group, the NfL levels were positively correlated with age (*p* < 0.03), as well as for TDP-43 (*p* < 0.01). In the MCI-AD group, the NfL levels correlated with age (0.47, *p* < 0.05), and the ratio Aβ42/Aβ40 (−0.36, *p* < 0.05) correlated negatively with age. In the groups of dementia-AD and FTLD, age did not correlate with plasma biomarkers.

## 3. Discussion

Recent studies about the highest efficiency of treatment in the early stages of AD have been published. It would involve relevant changes in the current clinical management of patients with mild cognitive impairment. For this, an easy and accessible diagnosis method is required, avoiding the CSF sampling. In this sense, novel techniques such as SIMOA show advantages in the detection of classical biomarkers in plasma and could help to validate new biomarkers [42].

In the present study, plasma biomarkers (Aβ42, Aβ40, p-Tau181, t-Tau, NfL, TDP-43, ratio t-Tau/Aβ42, ratio Aβ42/Aβ40) were determined by means of SIMOA. Among them, plasma p-Tau181 showed high accuracy in discriminating between healthy subjects from AD patients (MCI or mild dementia). These results are consistent with previous works in the literature [43,44,45]. Additionally, hyperphosphorylated Tau isoforms could be helpful for distinguishing stages of the AD spectrum (asymptomatic, MCI), so some authors postulated the use of p-Tau181 as a progression biomarker [19,46]. In this sense, in the present research, the highest levels of plasma p-Tau181 were obtained in the dementia-AD group, similarly to previous results [47]. However, another study in the literature did not show significant differences between dementia groups [48]. Related to neuropsychological performance, plasma p-Tau181 levels were negatively correlated with MMSE performance (score < 27 shows impairment). On the other hand, the determination of plasma p-Tau181 could discriminate between patients with cognitive impairment due to AD (MCI or dementia) and FTLD, as observed in previous works [40,49].

The plasma levels of Aβ42 and Aβ40 did not show statistically significant results discriminating between groups. Previous works showed similar results [50,51,52], indicating that the measurement of Aβ42 or Aβ40 individually could not show enough accuracy for AD diagnosis. Nevertheless, the measure of the Aβ42/Aβ40 ratio showed high accuracy distinguishing between controls and MCI due to AD, but also between the AD spectrum (MCI and dementia) and FTLD. These data are consistent with other studies, showing that a low Aβ42/Aβ40 ratio could discriminate between AD and controls [15,53]. In general, these results could be useful in patients in whom it may be challenging to perform a lumbar puncture or it is contraindicated because of cognitive decline or other characteristics, so that the potential plasma diagnosis could help in following treatment and prognosis. In the literature, some studies showed correlations between plasma Aβ42 and plasma p-Tau181 in AD [54], but the meaning of these correlations requires assessment.

The plasma NfL levels have been determined to detect neurodegeneration, as observed in previous studies [55,56,57]. In this sense, the present work showed high accuracy in distinguishing dementia-AD vs. control and FTLD vs. control. Moreover, plasma NfL levels had suitable sensitivity to discriminate between MCI-AD and dementia-AD. Additionally, plasma NfL levels correlated with worse neuropsychological performance (lower MMSE score and higher CDR score), constituting a potential neurodegeneration and dementia biomarker, as suggested in another study [27]. This finding could be helpful in progression studies. However, further research with larger sample size would be needed to validate these results and establish reliable cut-off values, indicating the risk of developing dementia due to AD.

Regarding TDP-43 levels, it is a molecule that has a critical role in RNA metabolism, and its presence in the form of cytoplasmic TDP-43 positive inclusion is a pathological mark of neurodegenerative diseases [58]. However, no significant correlations were found, nor were differences among the patients’ groups observed. According to other studies, it could be explained by the peripheral production of TDP-43, which may interfere in the specific disease measure [59,60]. Other studies showed that the levels of plasma TDP-43, as well as the CSF levels, were maybe not associated to the levels of cerebral pathology [61], and only some forms of TDP-43 in the brain have been detected in biofluid. Related to the neuropsychological test, TDP-43 levels correlated with the RBANS-DM score (score < 85 shows impairment).

Regarding CSF and plasma biomarkers, statistically significant correlations were observed for some biomarkers. In fact, p-Tau181 and NfL plasma levels correlated with their corresponding CSF levels. These findings mean that the process of neurodegeneration, measured with p-Tau181 (specific AD biomarker) and NfL (non-specific AD biomarker), could be detected in plasma. Moreover, the plasma Aβ42/Aβ40 ratio showed a significant correlation with the process of degeneration measured in CSF (t-Tau, ratio t-Tau/Aβ42), as well as with the specific AD diagnosis (CSF Aβ42). According to this, a recent study showed that the correlation between CSF and plasma biomarkers could be improved if the same technique was used for both sample type analyses [62].

In general, the simultaneous consideration of several plasma biomarkers levels could improve the early and specific diagnosis approach. So, some biomarkers panels have been evaluated in previous studies [63,64,65,66], showing some advantages in potential AD diagnosis. In fact, the use of only one plasma biomarker shows some limitations (non-specificity for AD, not enough sensitivity identifying AD stages). These inconveniences could be solved using the combination of some biomarkers for AD prediction. Similarly, the multivariate model developed in the present study could be helpful for discriminating between the controls and early AD (MCI-AD), with satisfactory accuracy (AUC 0.802), sensitivity (70%), and specificity (86%). Additionally, another developed model could help to discriminate between patients with cognitive impairment due to AD (MCI and dementia) from patients with FTLD (AUC 0.796). Nevertheless, few studies can be found in the literature in which the analyzed biomarkers could distinguish between AD and other pathologies (e.g., FTLD), and it could be explained by the only determination of the amyloid biomarkers [67] or by using another technique [68]. In this study, it is showed the convenience of combining different plasma biomarkers to increase the precision of the diagnosis, similar to other previous works suggesting similar recommendations for the diagnosis of AD [69] or to differentiate between AD and other neurodegenerative disease [31].

The main disadvantage of these plasma determinations, based on the ultra-sensitive analytical technique (SIMOA), is its high cost and the need for expensive commercial kits. In fact, nowadays, the standard CSF biomarkers determinations (Lumipulse^®^) are more cost effective than the plasma biomarkers determinations with SIMOA.

Limitations of this work include the small sample size, particularly in the FTLD and dementia-AD groups. However, the strength of this study was that all the groups were biologically classified from CSF biomarkers. Another limitation is the transversal design of the study and, therefore, the difficulty to make conclusions regarding AD spectrum. However, all the participants were neuropsychologically evaluated to identify the cognitive impairment degree as an approximation.

## 4. Materials and Methods

### 4.1. Participants and Samples Collection

This study was carried out in the unit of cognitive impairment (Hospital Universitari I Politècnic La Fe). The participants (*n* = 78) were aged between 52 and 78. They were classified according to the standard diagnosis criteria of the National Institute on Aging-Alzheimer’s Association, including CSF biomarkers and neuropsychological evaluation (CDR, MMSE, RBANS). The CDR (clinical dementia rating scale, it is composed of scale compromising global score (CDR-GS) and the sum of boxes score (CDR-SB)) is commonly used in staging cognitive impairment, and it could help discriminating across the different stages of disease [70], with well-established reliability and validity [71]. The MMSE (mini-mental state examination) is a score that evaluates three primary factors, i.e., verbal, memory, and constructional abilities, and it is commonly used for distinguishing patients with dementia from cognitively normal controls [72,73], because of their reproducibility and easily use. The RBANS (repeatable battery for the assessment of neuropsychological status) is a battery of tests that evaluated five functions by 12 subtests and whose scores could estimate, with reasonable accuracy, the cognitive deficits associated with AD and other pathologies [74,75,76]. In RBANS, the delayed memory domain (RBANS-DM) is the specific impaired domain in AD [77].

The participants were classified into healthy control group (HC, *n* = 22), mild cognitive impairment due to AD group (MCI-AD, *n* = 33), mild-dementia due to AD group (dementia-AD, *n* = 12), and frontotemporal lobar degeneration group (FTLD, *n* = 11). The HC group included participants with negative CSF AD biomarkers and a CDR value of ≤ 0.5. The MCI-AD group included patients with cognitive impairments, without daily living activities impairment, positive CSF biomarkers, and CDR = 0.5. The dementia-AD group included patients with positive CSF biomarkers and impairment of daily living activities (CDR = 1–2). Patients with moderate dementia (CDR 2–3) were not included. The FTLD group included patients who met the international behavioral variant FTLD criteria consortium (FTDC) and had negative CSF biomarkers [78]. For the control group (*n* = 17), only CDR and MMSE tests were applied, showing no clinical impairment and negative CSF biomarkers.

Samples (blood, CSF) were collected during routine clinical practice, and they were processed within 30 min and stored at −80 °C until analysis.

This study was approved by the Ethics Committee from Instituto de Investigación Sanitaria La Fe (Valencia, Spain) (reference number: 2020-079-1; date: 21 February 2020). All participants signed informed consent prior to their recruitment.

### 4.2. Equipment and Commercial Kits

The plasma determinations were carried out in Quanterix SR-X ™ equipment (Billerica, MA, USA), a platform based on SIMOA^®^ technology, following the manufacturer instructions. Specifically, this technology consists of paramagnetic particles coupled with antibodies designed to bind to specific targets (Aβ42, Aβ40, t-Tau, p-Tau181, NfL, TDP-43) in the sample. Kits for SIMOA determinations (Simoa^®^ Abeta 1-42, Abeta 1-40, human Tau proteins, human neurofilament light polipeptide, human phospho-Tau protein_v2.1, human TAR DNA-binding protein 43 elisa kits) were from Quanterix (Billerica, MA, USA).

The CSF determinations were carried out by chemiluminescence (CLIA) immunoassay (Lumipulse^®^ G, Fujirebio, Tokyo, Japan) in the clinical diagnosis service from Hospital La Fe.

### 4.3. Plasma Sample Treatment and Biomarkers Determination

Blood samples were obtained from venous puncture in a tube containing EDTA, they were centrifuged for 15 min at 1160× *g* and room temperature, and the plasma fraction was separated in a new tube. Cerebrospinal fluid samples (CSF) were obtained from a lumbar puncture, following a standard clinical routine, and they were centrifuged 10 min at 1200× *g* and 4 °C. Plasma and CSF samples were collected simultaneously for each participant, and they were stored at −80 °C until analysis.

The biomarkers determined in plasma and CSF samples were Aβ42, Aβ40, t-Tau, p-Tau181, neurofilament light (NfL), and TAR DNA-binding protein 43 (TDP-43). Plasma levels were determined using the SIMOA^®^ technology. All these assays used different capture and detector antibodies. Briefly, this procedure consisted of sample incubation with magnetic beans, which were conjugated with specific antibodies. Then, a secondary antibody and an enzyme were added, obtaining the immunocomplex (bean/bound protein/detection antibody). In the lector, each immunocomplex was captured by one individual well, and the lector detected the signal of one single molecule [79]. Finally, the biomarker concentrations were determined from the corresponding calibration curves, constructed from several calibrator points for each assayed peptide.

### 4.4. Statistical Analysis

The univariate statistical analysis was carried out using the SPSS software (version 22, IBM SPSS, Inc., Chicago, IL, USA). Numerical variables were expressed as median and interquartile range (IQR), and differences between groups were analyzed by the Mann–Whitney and Kruskal–Wallis tests. Categorical variables were expressed as a percentage, and differences between groups were analyzed by the Chi-square test. Correlations between plasma and CSF variables, as well as with other clinical variables, were analyzed by the Pearson correlation test. For all the analyses, statistical significance was established as *p*-value < 0.05.

The multivariate analysis was performed using R Statistical Software (version 4.2.1 (2022-06-23 ucrt) R, Vienna, Austria) [80], RStudio Integrated Development Environment (version 2022.12.0 (Build 353) R, Boston, MA, USA) [81], and mdatools R package (version 0.13.1, R, Aalborg, Denmark) [82]. Some multivariable regression models of partial least squares (PLS), based on the plasma biomarkers levels, to discriminate between AD and HC groups and between AD and FTLD groups, were developed. These analyses were performed with 6 independent predictor variables (Aβ42, Aβ40, t-Tau, p-Tau181, NfL, TDP-43) and 1 dependent response variable (AD, non-AD). All the variables were centered, and the predictors were auto scaled. The PLS models were fitted to the data from these six variables to calculate the individual probability of suffering from AD. The model was validated by K-fold cross-validation (k = 1).

The diagnosis potential of the developed PLS models was evaluated by means of the receiver operating characteristic (ROC) curve. The cut-off values were established as the highest sum of specificity and sensitivity from the ROC curve. The diagnosis indexes were calculated (AUC, sensitivity, specificity, positive predictive value (PPV), negative predictive value (NPV)), with their corresponding 95% confidence interval (CI). In addition, the regression coefficients (for centered and standardized data) were obtained to evaluate the different variables weight in each model; the corresponding model equations were provided, with the corrected coefficients to be applied directly to the raw data.

## 5. Conclusions

In this study, some potential plasma biomarkers (p-Tau181, t-Tau, Aβ42, Aβ40, NfL, TDP-43) were determined by means of a high sensitivity technique (SIMOA) to detect early and specific AD pathology, discriminating from healthy controls and FTLD patients. Specifically, p-Tau181 could be a promising plasma biomarker for specific AD diagnosis, since the early stages, and NfL could be a promising plasma biomarker for cognitive impairment degree identification. However, further studies with a large number of samples are needed to clinically validate these biomarkers and to establish reliable cut-off values in search of an established screening protocol for early and specific AD diagnosis in the general population.

## Figures and Tables

**Table 1:** Demographic and Clinical data from participants.

	HC (*n* = 22)	MCI-AD (*n* = 33)	Dementia-AD (*n* = 12)	FTLD (*n* = 11)	*p* Values (Kruskal–Wallis ^a^ Mann–Whitney ^b–f^)
Sex (female, *n*, (%))	8 (36%)	20 (61%)	6 (50%)	5 (46%)	0.364
Age (years, median (IQR))	70 (65–72)	67 (64–72)	70 (67–74)	63 (59–67)	0.029 ^a,^* 0.588 ^b^ 0.122 ^c^ 0.069 ^d^ <0.01 ^e,^* <0.02 ^f,^*
CSF Aβ40 (pg mL^−1^)	10979 (8872–15338)	11610 (9891–15793)	13844 (11950–19678)	13371 (8258–15610)	0.332 ^a^ 0.483 ^b^ 0.082 ^c^ 0.914 ^d^ 0.295 ^e^ 1 ^f^
CSF Aβ42 (pg mL^−1^)	1323 (1119–1686)	635 (500–767)	674 (494–758)	937 (814–1543)	<0.01 ^a,^* <0.01 ^b,^* 0.97 ^c^ <0.01 ^d,^* <0.01 ^e,^* <0.05 ^f,^*
CSF t-Tau (pg mL^−1^)	235 (185–308)	551 (361–765)	978 (659–1186)	351 (259–406)	<0.01 ^a,^* <0.01 ^b,^* <0.01 ^c,^* 0.053 ^d^ <0.01 ^e,^* <0.03 ^f,^*
CSF p-Tau181 (pg mL^−1^)	49 (33–62)	92 (50–117)	175 (121–208)	42 (37–70)	<0.01 ^a,^* <0.01 ^b,^* <0.01 ^c,^* <0.05 ^d,^* <0.01 ^e,^* 0.665 ^f^
CSF NfL (pg mL^−1^)	589 (523–775)	952 (744–1331)	1408 (1304–1992)	1607 (1190–4341)	<0.01 ^a,^* <0.01 ^b,^* <0.01 ^c,^* <0.01 ^d,^* 0.223 ^e^ <0.01 ^f,^*
CSF t-Tau/Aβ42	0.17 (0.13–0.21)	0.79 (0.46–1.06)	1.4 (1.28–1.85)	0.34 (0.27–0.48)	<0.01 ^a,^* <0.05 ^b,^* <0.01 ^c,^* <0.01 ^d,^* <0.01 ^e,^* <0.01 ^f,^*
CSF Aβ42/Aβ40	0.11 (0.104–0.122)	0.054 (0.048–0.059)	0.043 (0.037–0.05)	0.096 (0.064–0.109)	<0.01 ^a,^* <0.01 ^b,^* <0.05 ^c,^* <0.01 ^d,^* <0.01 ^e,^* <0.05 ^f,^*
MMSE (score, median (IQR))	28.5 (26–29)	25 (22.5–26.5)	16.5 (12.75–18.75)	19 (16–25)	<0.01 ^a,^* <0.01 ^b,^* <0.01 ^c,^* 0.093 ^d^ 0.118 ^e^ <0.01 ^f,^*
CDR (score, median (IQR))	0 (0–0.5)	0.5 (0.5–0.5)	1 (0.5–1)	1 (0.5–1)	<0.01 ^a,^* <0.01 ^b,^* <0.01 ^c,^* <0.05 ^d,^* 0.740 ^e^ <0.01 ^f,^*
RBANS-DM (score, median (IQR))	-	61 (51.5–68)	45 (10.75–50)	54 (47–62)	<0.01 ^a,^* <0.01 ^b,^* <0.01 ^c,^* 0.979 ^d^ <0.02 ^e,^* <0.02 ^f,^*

* *p* < 0.05. IQR: inter-quartile range; Kruskal–Wallis test: a; Mann–Whitney test: b = controls vs. AD (MCI-AD and dementia-AD), c = MCI-AD vs. dementia AD, d = MCI-AD vs. FTLD, e = dementia-AD vs. DLFT, f = controls vs. DLFT.

**Table 2:** Pearson’s correlations between CSF and plasma biomarkers.

		Plasma Biomarkers (*r*, p)							
		Aβ42	Aβ40	t-Tau	p-Tau181	NfL	Ratio Aβ42/Aβ40	Ratio t-Tau/Aβ42	TDP-43
CSF biomarkers (*r*, p)	Aβ42	0.102 (*p* = 0.38)	- 0.045 (*p* = 0.77)	−0.053 (*p* = 0.65)	- 0.441 (*p* < 0.01)	−0.008 (*p* = 0.95)	0.277 (*p* = 0.01) *	0.059 (*p* = 0.61)	0.14 (*p* = 0.21)
	Aβ40	0.136 (*p* = 0.32)	0.065 (*p* = 0.63)	−1.48 (*p* = 0.28)	0.094 (*p* = 0.48)	0.051 (*p* = 0.71)	0.082 (*p* = 0.54)	−0.144 (*p* = 0.28)	−0.035 (*p* = 0.79)
	t-Tau	−0.099 (*p* = 0.39)	−0.045 (*p* = 0.69)	−0.089 (*p* = 0.45)	0.611 (*p* < 0.01) *	0.138 (*p* = 0.234)	−0.27 (*p* = 0.02) *	−0.093 (*p* = 0.41)	−0.091 (*p* = 0.43)
	*p*-Tau181	−0.087 (*p* = 0.45)	−0.014 (*p* = 0.9)	−0.091 (*p* = 0.43)	0.649 (*p* < 0.01) *	0.066 (*p* = 57)	−0.297 (*p* < 0.01)	−0.13 (*p* = 0.26)	−0.035 (*p* = 0.79)
	NfL	−0.125 (*p* = 0.36)	−0.18 (*p* = 0.18)	−0.138 (*p* = 0.31)	−0.089 (*p* = 0.5)	0.86 (*p* < 0.01) *	0.088 (*p* = 0.51)	−0.03 (*p* = 0.82)	−0.17 (*p* = 0.9)
	Ratio Aβ42/Aβ40	−0.019 (*p* = 0.89)	0.032 (*p* = 0.81)	0.01 (*p* = 0.94)	0.064 (*p* = 0.63)	−0.083 (*p* = 0.54)	−0.037 (*p* = 0.78)	0.001 (*p* = 0.99)	−0.031 (*p* = 0.82)
	Ratio t-Tau/Aβ42	−0.13 (*p* = 0.32)	0.20 (*p* = 0.88)	−0.16 (*p* = 0.25)	0.605 (*p* < 0.01) *	−0.004 (*p* = 0.97)	−0.497 (*p* < 0.01) *	−0.225 (*p* = 0.08)	−0.22 (*p* = 0.09)

* *p* < 0.05.

**Table 3:** Plasma biomarkers levels obtained for each participants group.

(Median (IQR)) (pg mL^−1^)	HC (*n* = 22)	MCI-AD (*n* = 33)	Dementia-AD (*n* = 12)	FTLD (*n* = 11)	*p*-Value Kruskal–Wallis	*p*-Value Mann–Whitney
Aβ42	10.1 (6.76–11.97)	8.92 (7.08–10.8)	8.56 (7.91–10.65)	9.2(7.33–12.81)	0.378	0.481 ^a^ 0.63 ^b^ 0.75 ^c^ 0.88 ^d^ 0.46 ^e^ 0.45 ^f^
Aβ40	243 (207–278)	250 (213–281)	239 (216–289)	243 (192–305)	0.998	0.897 ^a^ 0.83 ^b^ 0.93 ^c^ 0.99 ^d^ 0.98 ^e^ 0.88 ^f^
t-Tau	2.86 (2.45–4.06)	3.61 (2.8–4.36)	3.32 (2.58–3.54)	3.00 (2.56–3.44)	0.373	0.255 ^a^ 0.87 ^b^ 0.96 ^c^ 0.21 ^d^ 0.15 ^e^ 0.65 ^f^
p-Tau181	1.44 (1.07–1.65)	2.68 (1.94–3.29)	3.42 (2.16–5.98)	1.51 (1.35–2.01)	0.01 *	<0.01 ^a,^* <0.01 ^b,^* 0.09 ^c^ 0.09 ^d^ <0.03 ^e,^* <0.01 ^f,^*
NfL	16 (12.21–20.06)	17.69 (14.87–26.34)	32.35 (16.61–41.49)	30.31 (25.21–51.68)	0.01 *	0.06 ^a^ <0.01 ^b,^* <0.01 ^c,^* <0.02 ^d,^* <0.01 ^e,^* 0.49 ^f^
TDP-43	136 (97–261)	154 (94–268)	253 (172–300)	199 (165–361)	0.53	0.47 ^a^ 0.12 ^b^ 0.08 ^c^ 0.12 ^d^ 0.08 ^e^ 1 ^f^
Ratio Aβ42/Aβ40	0.04 (0.034–0.044)	0.036 (0.033–0.041)	0.036 (0.034–0.037)	0.039 (0.036–0.043)	0.04 *	<0.02 ^a,^* 0.09 ^b^ 0.99 ^c^ 0.87 ^d^ <0.04 ^e,^* 0.06 ^f^
Ratio t-Tau/Aβ42	0.33 (0.25–0.45)	0.37 (0.31–0.54)	0.34 (0.28–0.43)	0.352 (0.2–0.34)	0.058	0.327 ^a^ 0.93 ^b^ 0.51 ^c^ 0.3 ^d^ 0.21 ^e^ 0.52 ^f^

* *p* < 0.05; a = HC vs. MCI-AD; b = HC vs. dementia-AD; c = HC vs. FTLD; d = MCI-AD vs. dementia-AD; e = MCI-AD vs. FTLD; f = dementia-AD vs. FTLD. IQR: Inter-quartile Range.

**Table 4:** Diagnosis indexes for the developed AD prediction models.

PLS Model	AUC (95% CI)	*p* Value	Sensitivity (%, 95% CI)	Specificity (%, 95% CI)	PPV (%, 95% CI)	NPV (%, 95% CI)
HC vs. MCI-AD	0.802 (0.685–0.918)	<0.01 *	69.7 (52.7–82.6)	86.4 (66.7–95.3)	88.5 (71.0–96.0)	65.5 (47.3–80.1)
HC vs. AD (MCI- + dementia)	0.809 (0.704–0.914)	<0.01 *	73.3 (59.0–84.0)	86.4 (66.7–95.3)	91.7 (78.2–97.1)	61.3 (43.8–76.3)
MCI-AD vs. FTLD	0.813 (0.687–0.938)	<0.01 *	75.8 (59.0–87.2)	81.8 (52.3–94.9)	92.6 (76.6–97.9)	52.9 (31.0–73.8)
AD (MCI-AD+ dementia-AD) vs. FTLD	0.796 (0.679–0.913)	<0.01 *	62.2 (47.6–74.9)	100 (74.1–100)	100 (87.9–100)	39.3 (23.6–57.6)

* *p* <0.05. AUC: area under the curve. PPV: positive predictive value. NPV: negative predictive value. CI: confidence interval.

**Table 5:** Regression coefficients for the variables in each PLS model.

	MCI-AD vs. HC	AD vs. HC	MCI-AD vs. FTLD	AD vs. FTLD
Aβ40	0.010	−0.001	−0.061	−0.022
p-Tau181	0.526	0.381	0.282	0.247
Aβ42	−0.110	−0.105	−0.154	−0.232
t-Tau	−0.029	0.032	0.155	0.098
NfL	0.162	0.220	−0.396	−0.413
TDP-43	−0.178	−0.117	−0.096	−0.075

**Table 6:** Corrected coefficients for the models’ equations.

	MCI-AD vs. HC	AD vs. HC	MCI-AD vs. FTLD	AD vs. FTLD
a	0.117	0.317	0.804	1.086
b	7.66 × 10^−5^	−4.22 × 10^−6^	−3.89 × 10^−4^	−1.33 × 10^−4^
c	0.252	0.117	0.122	0.063
d	−1.71 × 10^−2^	−1.65 × 10^−2^	−2.09 × 10^−2^	−3.10 × 10^−2^
e	−1.21 × 10^−2^	1.36 × 10^−2^	5.55 × 10^−2^	3.48 × 10^−2^
f	8.25 × 10^−3^	9.02 × 10^−3^	−7.99 × 10^−3^	−8.16 × 10^−3^
g	−1.31 × 10^−4^	−0.90 × 10^−4^	−0.96 × 10^−4^	−0.77 × 10^−4^

**Table 7:** Correlations between plasma biomarkers vs. neurocognitive performance and age.

r (p)	Aβ40 (pg mL^−1^)	Aβ42 (pg mL^−1^)	p-Tau181 (pg mL^−1^)	t-Tau (pg mL^−1^)	NfL (pg mL^−1^)	TDP-43 (pg mL^−1^)	Ratio t-Tau/Aβ42	Ratio Aβ42/Aβ40
Age (years)	0.49 (0.67)	−0857 (0.48)	−0.21 (0.86)	−0.52 (0.65)	−0.004 (0.97)	−0.134 (0.24)	−0.39 (0.74)	−0.297 (<0.01) *
CDR (score)	0.13 (0.25)	0.181 (0.12)	0.12 (0.3)	0.05 (0.69)	0.363 (<0.01) *	−0.07 (0.53)	−0.146 (0.2)	0.11 (0.34)
MMSE (score)	0.039 (0.75)	0.087 (0.43)	−0.39 (<0.01) *	0.17 (0.15)	−0.535 (<0.01) *	−0.002 (0.98)	0.12 (0.29)	0.585 (0.58)
RBANS-DM (score)	−0.15 (0.2)	−0.06 (0.618)	0.75 (0.52)	0.357 (<0.01) *	−0.09 (0.44)	0.335 (<0.01) *	0.331 (<0.01) *	0.087 (0.45)

r Pearson correlation. * *p* value <0.05.

## Data Availability

The data presented in this study are available on request from the corresponding author.
